# Shell-Less Egg Syndrome (SES) Widespread in Western Canadian Layer Operations Is Linked to a Massachusetts (Mass) Type Infectious Bronchitis Virus (IBV) Isolate

**DOI:** 10.3390/v10080437

**Published:** 2018-08-18

**Authors:** Aruna Amarasinghe, Shelly Popowich, Upasama De Silva Senapathi, Mohamed Sarjoon Abdul-Cader, Frank Marshall, Frank van der Meer, Susan C. Cork, Susantha Gomis, Mohamed Faizal Abdul-Careem

**Affiliations:** 1 Department of Ecosystem and Public Health, Faculty of Veterinary Medicine, University of Calgary, Health Research Innovation Center 2C53, 3330 Hospital Drive NW, Calgary, AB T2N 4N1, Canada; amarasinghearachchig@ucalgary.ca (A.A.); yaseshwari.desilvase@ucalgary.ca (U.D.S.S.); mohamedsarjoon.moham@ucalgary.ca (M.S.A.-C.); fjvander@ucalgary.ca (F.v.d.M.); sccork@ucalgary.ca (S.C.C.); 2 Department of Veterinary Pathology, Western College of Veterinary Medicine, University of Saskatchewan, Saskatoon, SK S7N 5B5, Canada; shelly.popowich@usask.ca (S.P.); smg127@mail.usask.ca (S.G.); 3 Marshall Swine and Poultry Health Services, 3831-Bay G-44 Ave, Camrose, AB T4V 3T1, Canada; frankm@cable-lynx.net

**Keywords:** infectious bronchitis virus, shell gland, shell-less egg, layer chicken, molecular epidemiology, spike protein gene, Western Canada

## Abstract

A disease with a sudden drop in egg production and shell-less eggs called, shell-less egg syndrome (SES) has been observed in Western Canada egg layer flocks since 2010. The etiology of this disease is not known. We hypothesize that SES is caused by an infectious bronchitis virus (IBV) strain since it is known that IBV replicates in the shell gland causing various eggshell abnormalities. In this study, we screened egg layer flocks, in the provinces of Alberta (AB) and Saskatchewan (SK), with and without a history of SES for the presence of IBV infection. During 2015–2016, a total of 27 egg layer flocks were screened in AB (*n* = 7) and SK (*n* = 20). Eighty-one percent of the screened flocks (*n* = 22) were positive for IBV infection. Thirty of these isolates were successfully characterized using molecular tools targeting the most variable *spike* (*S*) 1 gene. IBV isolates from this study clustered into three genotypes based on partial *S1* gene variability. The majority of the IBV isolates (70%) were Massachusetts (Mass) type, and the rest were either Connecticut (Conn) type or an uncharacterized genotype with genetic characteristics of Mass and Conn types. Since the majority of the IBV isolates included within the Mass type, we used a Mass type IBV isolate to reproduce SES in specific pathogen free (SPF) white leghorn chickens in lay. Further studies are warranted to investigate whether other IBV isolates can cause SES, to clarify the pathogenesis of SES and to develop a vaccine in order to prevent SES as observed in Western Canadian layer flocks.

## 1. Introduction

Since 2010 an increasing number of observations of transient shell-less egg production in layer operations located in Saskatchewan (SK), Canada was reported, and recently, this was also reported in Manitoba and Alberta (AB). These are sporadic outbreaks in layers of varying ages, during lay and affected flocks generally show a drop in egg production in addition to the production of shell-less eggs. This condition is known as “shell-less egg syndrome” (SES) and is thought to be caused by a strain of IBV since some strains of IBV are known to target the shell gland of the reproductive tract where the shell of the egg is formed [1]. IBV is known to cause egg-shell abnormalities in layers including soft thin shells [2], distorted shells [2], and discolored shells [3].

Egg production in laying chicken can be affected by a number of avian viruses including hepatitis E virus, Newcastle disease virus (NDV), low pathogenic avian influenza virus (LPAIV), avian encephalomyelitis virus (AEV), infectious bronchitis virus (IBV), and egg drop syndrome virus (EDSV). All these viruses except AEV can affect the internal and external quality of eggs [1,4,5,6,7,8,9,10] and only EDSV can lead to shell-less egg production under natural and experimental situations [10,11,12,13]. Notably, also IBV can replicate in various body compartments and organs [1,14,15], including the shell gland, causing various eggshell abnormalities, reduced egg production, and quality depending on the age at lay and the strain of the virus [9,14,16].

IBV is a host-specific respiratory pathogen, which belongs to the family *Coronaviridae*, however, thus far there are no reports that indicated IBV could cause shell-less eggs. Although no specific reproductive-tract-tropic IBV strains are known, some IBV strains can cause lesions in the reproductive tract and the respiratory tissues [7]. IBV replicates in the ciliated cells of the epithelial lining of the chicken oviduct including magnum, tubular shell gland, and shell gland pouch [7]. Disruption of these mucosal cells during an IBV infection could cause the chicken to lay eggs with shell abnormalities. Egg production reduction due to IBV infection generally is between 3 and 10% but in exceptional cases can be as high as 50% [9]. Also, it has been estimated that chickens that recover from IBV infection may continue to produce about 10% fewer eggs compared to their expected normal production levels over the laying period [9].

The IBV genome undergoes mutations and recombination which can lead to changes in virulence and disease pathogenesis [17,18,19]. The *spike protein (S) 1* gene is the most variable and the main inducer of virus-neutralizing antibodies, while *S2* is relatively conserved [18,20,21,22,23]. Therefore, sequencing of the *S1* gene is the foremost technique that is used to differentiate IBV strains into various genotypes [17,20,21,22,23,24,25]. IBV strains circulating in Canada were characterized [21,25,26,27] through the analysis of a 505-nucleotide fragment of the *S1* gene obtained during IBV infection outbreaks in Ontario, Quebec, British Columbia, Nova Scotia, and Newfoundland and Labrador. The characterized IBV isolates of these Canadian provinces belonged to nine genotypes [21]. Further, in this study, the IBV strain 4/91 was found to be endemic to Ontario. Similarly, RFLP analysis of three field isolates from an IBV outbreak of a broiler chicken flock in Joliette in the province of Quebec, Canada using full-length *S1* gene amplification, detected a new phylogenetic cluster [21]. A 600-nucleotide fragment of the *S1* gene was used for characterization of five field isolates from Ontario, Canada, and this study reported the amino acid similarity of 55 to 71% to the M41 reference and MILDVAC-Ma5 vaccine strains [25]. There has been limited work done on the molecular nature of the IBV strains circulating in the Western part of Canada. This study aimed to determine whether IBV is associated with egg layer flocks affected with SES in Western Canada. The additional objectives were to isolate and molecularly characterize these IBV isolates and to reproduce SES experimentally with aiming to establish the etiology of SES.

## 2. Materials and Methods

### 2.1. Ethics

Ethical approval to carry out this study was obtained from the Health Science Animal Care Committee (HSACC) of the University of Calgary, protocol number AC15-0140, permission date (30 October 2015).

### 2.2. Eggs and Chickens

Fertile specific-pathogen-free (SPF) eggs and chickens were purchased from the Canadian Food Inspection Agency (CFIA).

### 2.3. Collection of Samples

Trachea, lung, kidney, uterus, and cecal tonsil (CT) samples were obtained from layer farms with and without a history of SES in the Western Canadian provinces of SK (*n* = 20) and AB (*n* = 7) from the beginning of 2015 to the end of 2016. In SK, 8 out of 12 flocks had a history of SES. In AB, 4 out of 7 flocks had a history of SES. The production-related data along with IBV serology results of the observed layer flocks are given in the Supplementary Table S1. The sampling was done with assistance from the SK Poultry Extension Program and, in AB, with the assistance of Marshall Swine and Poultry Health Veterinary Services, Camrose, AB. Upon collection, the samples were transported to the laboratory on dry ice as an overnight expedited shipment and stored at −80 °C until further processing.

### 2.4. Screening of Samples and Sequencing of IBV Partial S1 Gene

To identify IBV positive samples, a real-time polymerase chain reaction (PCR) targeting the conserved *nucleoprotein* (*N*) gene was used. The real-time PCR positive samples were subjected to conventional PCR assay targeting partial *S1* gene. Then, the samples that were negative in partial *S1* conventional PCR assay were propagated in 9–11 days old SPF embryonated eggs. After each round of propagation, collected allantoic fluid was subjected to a conventional PCR assay to amplify the partial *S*1 gene for sequencing.

#### 2.4.1. Tissue Homogenization

A piece of tissue (40 mg) was homogenized in 1 mL of cold PBS using a Pro200 Power homogenizer (Diamed, Mississauga, ON, Canada) on ice. The tissue homogenates were centrifuged at 1000× *g* at 4 °C for 10 min and the supernatant was stored at −80 °C until further processing.

#### 2.4.2. RNA Extraction and Complementary (c)DNA Synthesis

Total RNA was extracted from 250 µL of tissue homogenate using the Trizol LS^®^ reagent (Ambion, Invitrogen Canada Inc., Burlington, ON, Canada) following the manufacturer’s protocol. The RNA concentration was determined using a Nanodrop 1000 spectrophotometer at 260 nm wavelength (Thermo Scientific, Wilmington, DE, USA) and the RNA quality was determined using the Nanodrop based on the 260:280 UV absorbance ratio. About 2000 ng of extracted RNA was converted to complementary DNA (cDNA) using random primers (hexamers of d(N)6) (High Capacity Reverse Transcription Kit™, Applied Biosystems, Invitrogen Canada Inc., Burlington, ON, Canada). Also, a ‘no reverse transcriptase’ (NRT) control was included in the cDNA conversion step to detect cDNA contamination.

#### 2.4.3. Real-Time PCR Assay

Previously published real-time PCR assay was used to amplify a 200 nucleotide fragment of the IBV-*N* gene using primers: Fw-5′GACGGAGGACCTGATGGTAA3′ and Re-5′CCCTTCTTCTGCTGATCCTG3′, as described previously [28]. Melt curve analysis was performed to assess the specificity of reactions. Melting curve analysis was done between 65 °C to 95 °C with an increment of 0.5 °C at every 5 s. The sensitivity of the real-time PCR assay was determined as 1000 copies per 20 ng of cDNA, which gives an average cycle threshold (Ct) value of 37 [28].

#### 2.4.4. IBV Propagation in SPF Eggs

Briefly, tissue homogenates of *N*-gene real-time PCR positive samples that were negative in partial *S1* conventional PCR were passaged in nine days of age embryonated SPF chicken eggs and PBS was used as a negative control. Embryos were inoculated with homogenates originating from tissue samples in the allantoic cavity. Allantoic fluid was harvested at three days post-infection (dpi) and stored at −80 °C in aliquots of 0.5 mL.

#### 2.4.5. Conventional PCR Assay Targeting Partial IBV *S*1 Gene

A conventional two-step reverse transcriptase (RT)-PCR assay was carried out to amplify a fragment of 620 nucleotides of the IBV *S1* gene (a portion encoding a stretch of *S*1 N terminal domain, amino acids 26-231) using the primers Fw-5′GTKTACTACTAYCAAAGTGCCTT-3′ and Re-5′GCATGCWARCAARCCTCTAGG-3′ [21]. Both positive (M41 virus strain) and negative (water) controls were evaluated in the reactions. PCR amplicons were evaluated on a 1% agarose gel with a 1 kb DNA marker. DNA bands with the correct size of 620 bp were purified using an E.Z.N.A.^®^ gel purification kit (Omega Bio-Tek, Norcross, GA, USA) following the manufacturer’s instructions.

#### 2.4.6. Molecular Characterization of IBV Isolates

Sanger sequencing was performed at the Center for Applied Genomics (Hospital for SickKids, Toronto, ON, Canada) for both forward and reverse directions. The resulting sequences were visually checked. Sequences were manually edited and proof-read with reference to IBV M41 (GenBank accession: AY851295). Both forward and reverse contigs were de-novo assembled into one contig using Geneious assembler (Geneious^®^ 10.1.3, Biomatters Ltd., Auckland, New Zealand) with the highest sensitivity recommended for Sanger sequencing products. The aligned sequences were trimmed at both ends to the size of the shortest sequence read obtained. The resulting partial IBV *S1* nucleotide sequences (508 nucleotides) from the current study were deposited in the GenBank repository with the accession numbers ranging from MH509448 to MH509477 and were aligned with the published sequences of IBV reference strains and serotypes including Canadian strains [21] using the Clustal Omega 1.2.3 package with standard settings (Geneious^®^ 10.1.3); mBed cluster size was set to 100. Forty IBV sequences that were retrieved from the NCBI GenBank database using the nucleotide BLAST^®^ search tool representing common serotypes, vaccine strains and Canadian field IBV strains are listed in Supplementary Table S2.

Aligned nucleotide sequences were used to construct an unrooted phylogenetic tree by Randomized Accelerated Maximum Likelihood (RAxML) method [29] using the GTR Gamma nucleotide model and Rapid Hill-climbing algorithm. The accuracy of the tree branches was analyzed with 1000 bootstrap replicates while Parsimony random seed is set to 1 (Geneious^®^ 10.1.3; Biomatters Ltd., Auckland, New Zealand). Possible clustering of new IBV isolates was determined using the generated phylogeny along with similarities and dissimilarities to the reference serotypes used. Translate function of the Geneious software was used along with open reading frame (ORF) 1 following the standard genetic code. Translated amino acid sequences were realigned using the Global alignment with Blosum62 cost matrix. The RAxML phylogenetic tree was generated using RAxML plugin version 8.2.7 under the protein model GAMMA BLOSUM62 with Rapid bootstrapping and search for best-scoring ML tree with 1000 bootstrap replicates, with parsimony random seed is set to 1 [30]. Further separate analysis was performed with sequences from a total of 42 Canadian IBV isolates, including the 30 IBV strains from Western Canadian layer flocks with and without a history of SES isolated in the present work. The 12 additional isolates were recovered from Eastern parts of Canada and have been described previously [27].

### 2.5. Reproduction of SES Using Selected Mass Type IBV Isolates

#### 2.5.1. Pilot Experiment

Since 70% of IBV isolates originated in our survey were Mass type, we hypothesized that SES is likely to be caused by a Mass type IBV isolate originating from Western Canada layer flocks. The Mass type IBV isolates 15AB-01 (originating from AB) and 15SK-02 (originated from SK) were selected for experimental reproduction of SES. Before being used, the inoculums of 15AB-01 and 15SK-02 were screened for the presence of LPAIV [31], EDSV [32], and NDV [33] using real-time PCR assays.

Twenty-four-week old white leghorn SPF chickens in lay were obtained (*n* = 4) and allowed to acclimatize for 2 weeks in high containment poultry isolators. The feed was a commercial layer ration, which contained 17% of calcium and birds were provided 12-h light/dark cycle a day throughout the experimental period. At 26 weeks of age, 1 and 2 chickens were infected with the IBV isolate 15AB-01 and 15SK-02, respectively, with a dose of 1 × 10^6^ EID_50_ per bird under isoflurane anesthesia. A total volume of 400 µL per bird was used with 250 µL intratracheally, 100 µL intranasally, and 50 µL via the intra-ocular route. One chicken was treated as an uninfected control (phosphate buffered saline or PBS). Daily egg production was noted and checked for any shell abnormalities for 12 dpi.

#### 2.5.2. Experimental Reproduction of SES Using 15AB-01 Mass Type IBV Isolate

Six 18-week old SPF laying chickens were obtained from the CFIA, Ottawa, ON and transferred to high containment poultry isolators. Then the chickens were allowed for a 7-week period of acclimatization and stabilization of egg production with access to ad libitum feed and water. The feeding and lighting were done as indicated in the pilot experiment. At 25 weeks of age, 3 chickens were infected with the Mass type IBV field isolate (15AB-01) with a dose of 1 × 10^6^ EID_50_ per chicken under isoflurane anesthesia as indicated in the pilot experiment. Three chickens were treated with PBS as uninfected controls. Daily egg production was noted and checked for any eggshell abnormalities for 21 dpi. Tracheal and cloacal swabs were taken at 3, 7, 12, 15, 18, and 21 dpi and stored at −80 °C. At 21 dpi, chickens were euthanized and tissue samples were taken from the lung, trachea, CT, kidneys and ovary, isthmus, magnum, and shell gland of the reproductive tract into 1.7 mL centrifuge tubes containing 1 mL of RNASave^®^ (Biological Industries, Beit Haemek, Israel) and stored at −20 °C. The tissues were also collected into 10% neutral buffered formalin for histological observations. Chickens were also bled at 3, 7, 15, 18, and 21 dpi into plain tubes containing no anticoagulants. Blood tubes were left on the bench at room temperature for 30 min and centrifuged at 2000× *g* for 10 min at 4 °C, the serum was separated, and aliquots were stored at −80 °C until further analysis.

### 2.6. Histology

Formalin-fixed terminal tissue samples (21 dpi) were processed at the Diagnostic Services Unit of the University of Calgary’s Faculty of Veterinary Medicine including staining of sections with stained hematoxylin and eosin (H & E). Sections were examined under the light microscope and photomicrographs were taken under 40× magnification.

### 2.7. IBV Antibody Titer Determination

Serum anti-IBV antibody titers were determined using the IDEXX IBV antibody test kit (IDEXX Laboratories, Westbrook, ME, USA) following the manufacturer’s instructions. The plates were read at 650 nm absorbance using SpectraMax M2 microplate reader (Molecular Devices, Sunnyvale, NS, Canada).

### 2.8. Data Analyses

#### 2.8.1. IBV Genome Load Quantification

IBV RNA copies per 200 ng of cDNA were estimated based on the standard curve generated using a serial dilution of IBV plasmid.

#### 2.8.2. Statistical Analysis

Geometric mean IBV ELISA titers of the surveyed flocks with and without a history of SES were compared using student’s *t*-test. The Chi-square test with Yates correction was used to compare the total marketable egg production between the infected and uninfected control groups during the observation period. The Fisher’s exact test was used to compare the difference in marketable egg production between the infected and uninfected control groups at each day. IBV genome loads of the tracheal and cloacal swab samples at different time points were analyzed using Two-way ANOVA followed by a Bonferroni post-hoc test. IBV genome loads between tissues collected at 21 dpi were compared with One-way ANOVA followed by Tukey’s multiple comparisons. All statistical tests were performed using the built-in statistical analysis feature of the GraphPad™ Prism 7 (La Jolla, CA, USA). Differences were considered significant at *p* < 0.05.

## 3. Results

### 3.1. Background of the Screened Poultry Flocks with and without a History of SES

The relevant background data including IBV vaccination history and IBV specific antibody titers of the surveyed layer flocks with and without a history of SES are given in the Supplementary Table S1. The average age of the surveyed flocks was 39.68 weeks (range 18–70 weeks) and the average flock size was 9692.41 (range 5000–45,288 chickens per flock). We could collect data of egg production drop in flocks with a history of SES and the average drop in egg production observed was 21.67% (range 7–46%). The vaccination history data were available for SK flocks (Supplementary Table S1) and the data suggest that the surveyed flocks have been vaccinated against IBV and the average IBV ELISA titer (geometric mean) was 3918.34 (range 342–13,052). We did not see a difference in IBV ELISA titer between flocks (*p* = 0.7230) that had a history of SES (4147.86 ± 1067.39) and the flocks without a history of SES (3596.00 ± 918.13).

### 3.2. Sample Screening for the Detection of IBV Genome

Total RNA was extracted from 602 tissue samples (lung, trachea, CT, kidney, and uterus) belonging to 27 farms with and without a history of SES and of which 79 samples (13% of the examined samples) were positive in the IBV *N* gene real-time PCR assay. IBV positive samples originated from 22 farms (81% of the examined flocks), and 4/7 and 18/20 farms were positive for IBV genomes in AB and SK, respectively (Table 1). Overall, 10 out of 12 farms with a history of SES were positive for IBV, while 13 out of 15 farms without a history of SES were positive for IBV. In AB and SK, 3 out of 4 and 7 out of 8 farms with a history of SES were positive for IBV, respectively. Eight out of 10 and 5 out of 5 farms without a history of SES in AB and SK respectively were positive for IBV genomes (Table 1).

### 3.3. Egg Propagation, IBV S1 Gene Amplification, and Sequencing

**Figure 1:**
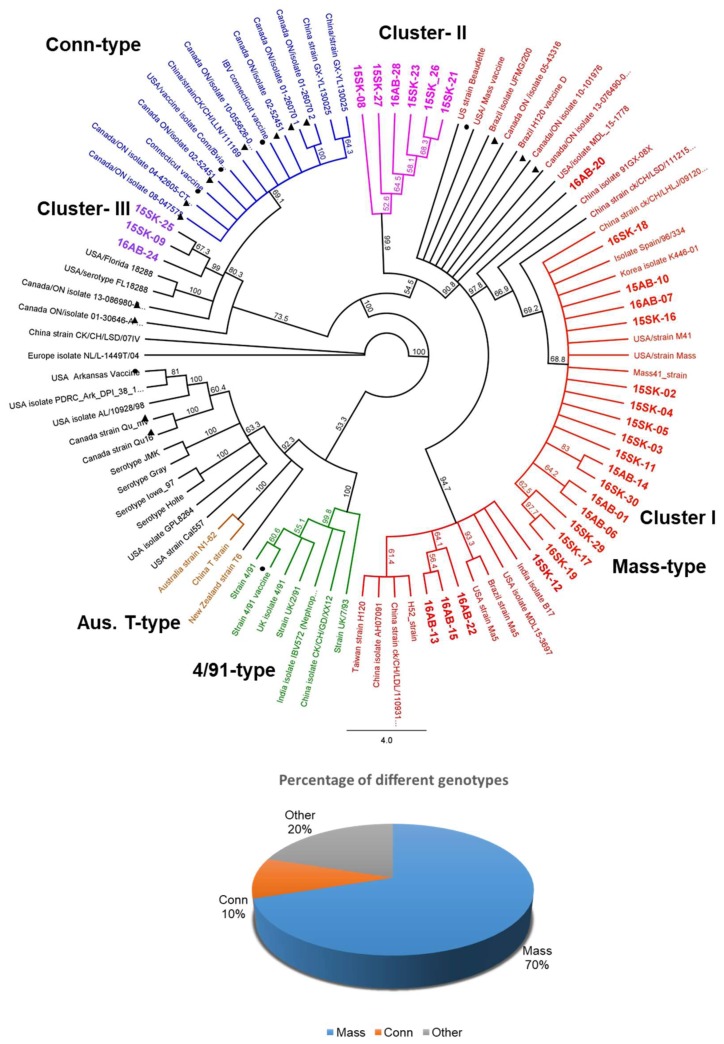
Nucleotide phylogenetic tree of IBV *S1*. Maximum likelihood phylogenetic tree inferred based on the 508-nucleotide fragment of the *S1* nucleotide sequence. The resulting cladogram of the 30 strains of this study including 64 reference sequences is shown along with bootstrap values (current study isolates in bold; vaccine reference strains are marked with ●; previous Canadian strains are marked with ▲; IBV reference clusters and sub-clusters are color-coded). Scale bar indicates the percentage of sites changing along each branch. The pie chart shows the percentage of current IBV strains, which represent each genotype.

**Figure 2:**
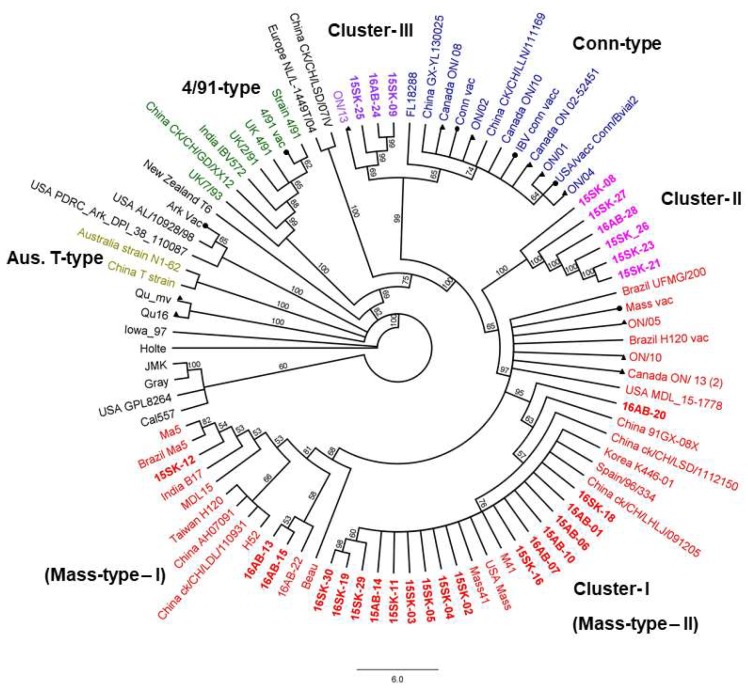
Maximum likelihood phylogenetic tree of IBV *S1* based on deduced amino acid sequences. The bootstrap support is shown for each node (current study strains are color-coded and depicted in bold; vaccine strains are marked with ●; Canadian field strains are marked with ▲; IBV reference clusters and sub-clusters have been color-coded). Scale bar indicates the number of changes inferred as having occurred along each branch. Scale bar indicates the percentage of sites changing along each branch.

Of the 79 real-time PCR positive samples, 21 were positive for partial *S1* conventional PCR without being propagated in eggs. Nine real-time PCR positive samples that were negative for partial *S1* conventional PCR were egg passaged. About 6–7 cycles of repeated egg passages were required for these 9 samples to obtain adequate IBV titers to amplify partial *S1* gene by conventional PCR. Based on the phylogram drawn using the IBV *S1* short fragment nucleotide sequence, 21 (70%) IBV isolates clustered with the Mass type IBV. Three (10%) IBV isolates formed a sub-cluster within the Conn type while 6 (20%) isolates formed a separate uncharacterized cluster (Cluster II in Figure 1) in between Mass and Conn types without a reference strain (Figure 1). These 6 isolates had 18 single nucleotide polymorphism (SNPs) compared to the IBV Mass strain consensus sequence. Out of them, 13 were nonsynonymous substitutions. The 6 isolates in cluster II shared a maximum nucleotide identity of 96.4% with the IBV Mass strain and 95.4–96.6% with the Canada Ontario isolates and the Conn serotype isolates. However, the current IBV isolates were clearly separated from the vaccinal Mass and Conn strains (Figure 1 and Figure 2). Deduced % amino acid similarities of the current field isolates with the Canadian and USA reference strains ranged from 65.3 to 100%. The highest % amino acid similarity that the current study isolates shared with the Mass vaccinal strains was 98.2% (Supplementary Figure S1).

**Figure 3:**
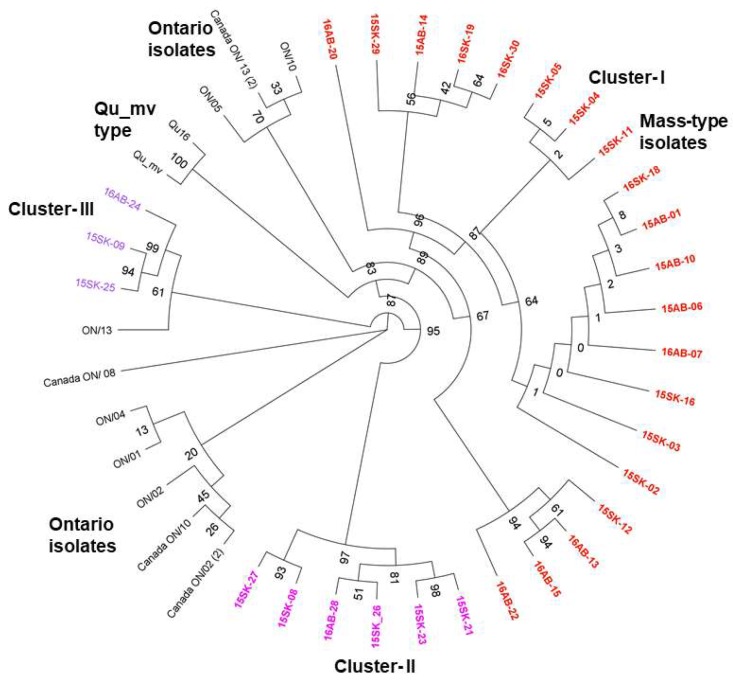
Phylogenetic tree of 42 Canadian IBV isolates: Maximum likelihood phylogenetic tree inferred based on the deduced amino acid sequences of the partial *S1* gene is shown. The bootstrap support for each node is shown (current SES isolates are color-coded and depicted in bold).

A phylogenetic tree comparing 42 Canadian strains including current IBV strains are illustrated in Figure 3. All the current isolates were different from Canadian specific IBV strains, Ontario types, and Qu-mv types (amino acid similarity ≤96% and 69.2%, respectively). The amino acid similarity with the Mass vaccine strain ranged from 98.2% (16AB-20) to 88.5% (16AB-24) (Supplementary Figure S1). According to the inferred protein phylogenetic tree, the current isolates, 15SK-09, 15SK-25, and 16AB-24 formed a separate cluster from the Conn genotype (Figure 2). These 3 isolates were related to Canada Ontario strains but have emerged from earlier branches. Similarly, 6 of the current isolates (15SK-08, 21, 23, 26, 27, and 16AB-26) formed a branch deviating from the other Mass type isolates. Three of the current isolates were related to China and Korean Mass type isolates while the majority (9) of the current isolates were clustered with the prototype M41 strain. Many of the amino acid sequences deduced from the 30 partial *S1* gene sequences were identical; there were 8 unique amino acid sequences (only the current isolates representing the 8 unique amino acid sequences are shown in Supplementary Figure S1); the 6 isolates in the cluster II were similar. Also, the 3 isolates in cluster III were similar. The Mass type isolates, 15SK-12, 16AB-13, 16AB-15, 16AB-20, and 16AB-22 carried differences to the rest of the Mass-type isolates, which were mostly similar. In Farm #2, isolates 16AB-13, 16AB-15, 16AB-20, and 16AB-22 were unique Mass type isolates and 16AB-28 belongs to Cluster II. In Farm #22, 15SK-09, and 15SK-25 were Conn type isolates whereas 15SK-17 was a Mass type. In Farm #1, 8, 19, and 23, which had multiple isolates, the isolates were similar within each farm.

### 3.4. Experimental Reproduction of SES

#### 3.4.1. Pilot Experiment

The inoculums of isolates 15AB-01 and 15SK-02 were free of genomes of LPAIV, EDSV, and NDV as indicated by real-time PCR assays.

Only the chicken infected with IBV isolate, 15AB-01, produced a shell-less egg at 3 dpi (Supplementary Figure S2). Then the chicken infected with IBV isolate 15AB-01 did not lay eggs between 4–5 dpi, layed a smaller egg at 6 dpi, and then produced regular eggs until 12 dpi. The chickens infected with 15SK-02 IBV isolate did not produce any shell-less eggs but produced a defective shell egg on 10 dpi and no regular eggs after 6 dpi except 8 dpi. However, the uninfected control chickens did not produce eggs on 5 dpi and between 8–12 dpi.

#### 3.4.2. Egg Production Following Infection with 15AB-01 Mass Type IBV Isolate

**Figure 4:**
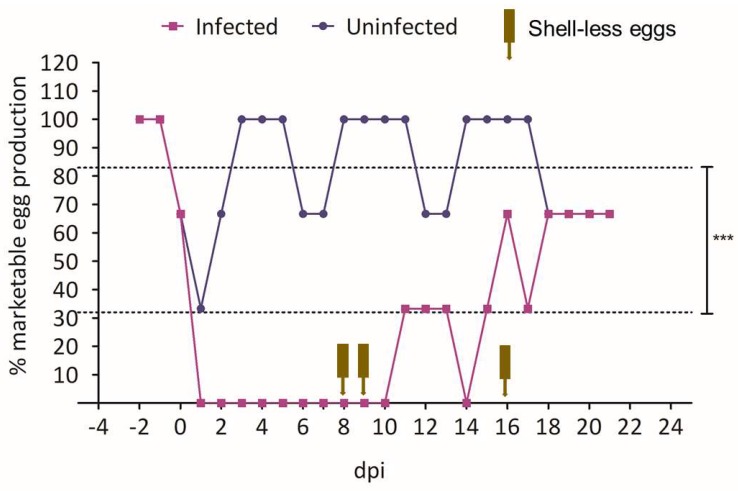
Egg production following infection with Mass type Western Canadian IBV isolate, 15AB-01. The percent daily egg production by the two groups of birds (only regular eggs are included in the percentage) is shown. Arrows indicate production of shell-less eggs. Dotted lines indicate the mean egg production values for the two groups; *** = *p* < 0.001—indicates a significant difference in total marketable egg production between infected and control groups during the observation period as analyzed by Chi-Square test. The difference in marketable egg production each day is binary (marketable eggs or not), as such, we used the Fisher’s exact test to see the difference in egg production each day.

All the chickens infected with 15AB-01 IBV isolate did not produce regular eggs for 10 dpi. The IBV infected group of chickens produced 2 and 1 shell-less eggs on 8 and 9 dpi, respectively. Thereafter for 3 days, the infected chickens produced 1 regular egg per day. On 16 dpi, the infected chickens produced 2 regular eggs and 1 shell-less egg. From 18 dpi until the end of the study (21 dpi), the infected chickens produced 2 regular eggs per day (Figure 4). In comparison, the uninfected control chickens produced 2–3 regular eggs daily throughout the study period except for 1 dpi (Figure 4). There was a highly statistically significant difference in total marketable egg production between the infected and control groups during the observation period (*p* = 0.0001). However, the difference in marketable egg production between the two groups at each day was not statistically significant (*p* > 0.05).

#### 3.4.3. IBV Genome Loads Following Infection with 15AB-01 Mass Type IBV Isolate

**Figure 5:**
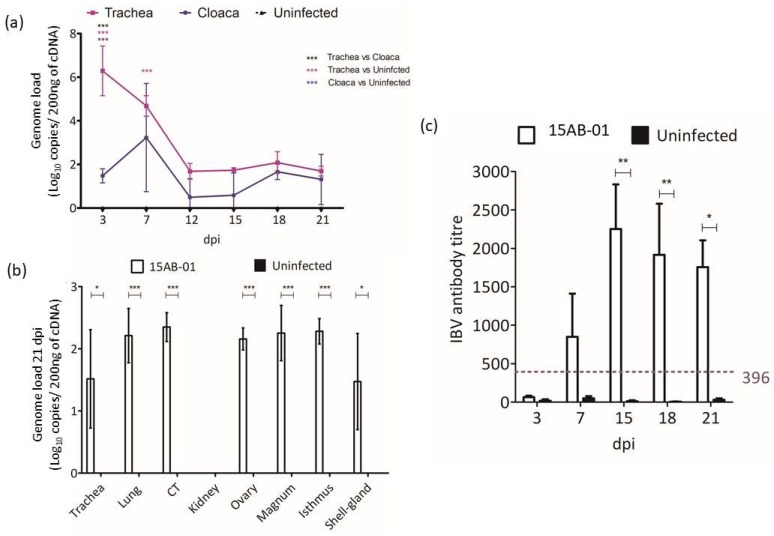
IBV genome load and anti-IBV antibody response following infection with Mass type Western Canadian IBV isolate, 15AB-01. Absolute genome loads for the tracheal and cloacal (**a**), and various tissue samples (**b**) are shown in the graphs. Absolute serum anti-IBV antibody titer is shown in graph (**c**) (dotted line indicates cut-off test value). Error bars indicate standard error of the mean (SEM). (key to statistical significance: * *p* < 0.05, ** *p* < 0.01, and *** *p* < 0.001).

The uninfected controls showed no detectable levels of the IBV genome in any tissue or tracheal and cloacal swabs throughout the observation period. Figure 5a displays the IBV genome load as detected in tracheal and cloacal swabs of IBV infected chickens. At 3 dpi, the IBV genome loads in the tracheal swabs of IBV infected chickens were significantly higher than those in cloacal swabs (*p* < 0.0001).

IBV genome loads in tissues collected at 21 dpi can be found in Figure 5b. Kidneys of the infected chicken had no detectable levels of IBV genome loads. The rest of the examined tissues from the infected chickens showed low IBV genome loads. The IBV genome loads in CT were significantly higher than those in the kidney (*p* < 0.05).

#### 3.4.4. Antibody Response Following Infection with 15AB-01 Mass Type IBV Isolate

The antibody response in serum collected from IBV infected and control chickens is illustrated in Figure 5c. The serum antibody response became significantly higher in infected chickens compared to controls from 15 dpi (*p* < 0.01) onwards.

#### 3.4.5. Histological Changes in the Shell Gland Following Infection with 15AB-01 Mass Type IBV Isolate

**Figure 6:**
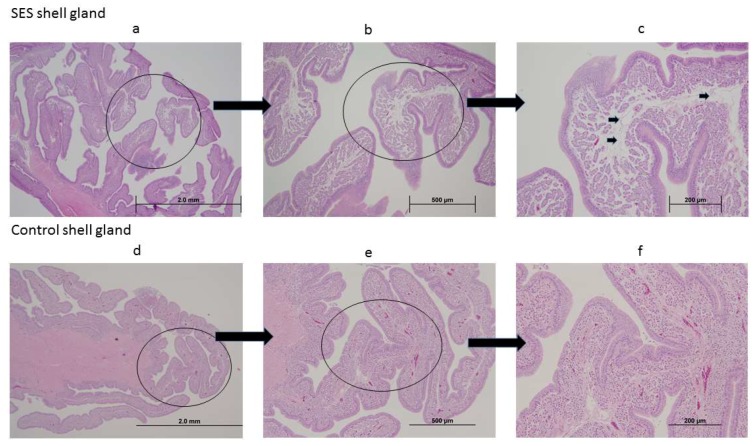
Histological changes following infection with Mass type Western Canadian IBV isolate, 15AB-01. Images (**a**–**c**) are representative images of the shell gland from IBV infected chicken, and the images (**d**–**f**) are representative images of the shell gland from uninfected chicken. Edema is shown using arrows in the image (**c**).

The mucosal folds of the shell gland of 2 out of 3 infected chickens showed edema (Figure 6a–c) when compared to uninfected controls at 21 dpi (Figure 6d–f). The mucosal folds of the shell gland of the remaining infected chicken showed no lesions and were similar to the mucosal folds of shell glands of the uninfected controls.

## 4. Discussion

The described studies led to several major findings. First, IBV infections in Western Canada layer flocks are caused mainly by strains related to Mass and Conn. Second, we did not observe a difference in IBV infection between flocks with and without a history of SES in Western Canada. Third, the majority (70%) of IBV strains detected in layer flocks were Mass-type IBV. Fourth, the field IBV isolates that formed a separate cluster II did not align with any of the known reference sequences. Fifth, SES recently observed in Western Canada layer flocks is linked to a Mass type IBV isolate.

The IBV strains detected in AB and SK are unknown until now and the current investigation records IBV strains isolated from layer flocks in these two provinces of Canada. Unlike in other Canadian provinces, the IBV strains observed in Western Canada are mainly of Mass and Conn genotypes. However, further studies are required in order to classify these IBV isolates serologically. Some data on the incidence and distribution of IBV isolates have been published in Canada, but there was limited information available about strains in the Western regions, notably AB and SK. In Quebec, between 1976 and 1980, 24 isolates of IBV have been characterized based on serum neutralization assay [34] which included Conn, Holland, and SE-17 types. In 1987, five IBV isolates recovered from unvaccinated layer flocks with a history of respiratory illness and increased mortality in Ontario have been recorded as Mass type based on serum neutralization assay [35]. Between 1996–1999, in Quebec and New Brunswick, three IBV isolates have been characterized based on sequencing [27] which recorded variants of IBV due to recombination of Mass vaccine and Ark type strains. In 2002, a variant of DE/072/92 with clinical signs of respiratory involvement and a nephropathogenic variant of PA/1220/9 had been isolated in Ontario poultry flocks [25,26]. During 2000–2003, IBV variant strains had been isolated in poultry flocks in Ontario, Quebec, British Columbia, Nova Scotia, and Newfoundland and Labrador [21] which recorded nine IBV genotypes. These nine genotypes included Canadian variant virus (strain Qu_mv, the classic and vaccine-like viruses, Conn and Mass) and US variant-like virus strains (California 1734/04, California 99, CU_82792, Pennsylvania 1220/98, and Pennsylvania Wolg/98) and non-Canadian, non-US virus (strain 4/91). During 2014–2017, involving samples from Ontario and Quebec, 10 genotypes of IBV have been identified which included Mass, Conn, NT, PA Wolg98, Qu-MV, 4/91, CA1737, DE072, DMV, and GA2015 genotypes [36].

IBV infection in very young chickens can cause permanent damage to the oviduct, resulting in the cystic oviduct and fully grown false layer chickens [37]. IBV infection in laying chickens typically results in low egg production and eggshell defects [38]. For example, in an experiment involving 77-week-old white leghorn chickens, it has been shown that an Ark strain of IBV leads to 15% lower egg production, lower eggshell weight, poor egg internal quality, and fewer grade A eggs and more grade B and C eggs when compared to the controls [16]. The egg production losses generally are 3–10%, but it could be as high as 50% in some IBV outbreaks [39]. Also, it has been estimated that birds that recover from IBV infection may continue to produce about 10% fewer eggs than normal production levels over the laying period [9]. However, we could not find any literature that indicates that IBV infection in chickens in lay leads to shell-less eggs. Similar to EDSV, the IBV isolate, 15AB-01, that led to shell-less eggs, also showed evidence of IBV infection in the reproductive tract including shell gland [12]. In the IBV infected (15AB-01 isolate) chicken edema in the shell gland mucosa was evident and similar occurrence of edema had been detected following infection with EDSV [40]. Although the latter study also recorded other histological changes such as degeneration and desquamation of the ciliated epithelial cells, atrophy of the uterine glands and heterophil cell infiltration in the shell gland, we did not observe these changes at 21 dpi. However, we recognize that our experiment was not designed to study these pathological changes in the early stages of 15AB-01 IBV isolate infection and further studies are required to investigate the pathological consequences of the SES IBV in the shell gland of the chickens in lay. It is also important to note that the current SES reproduction study did not include a control group with prototype IBV, M41, in the animal experiment, and this is a limitation that we consider in concluding our study findings.

In conclusion, we found that the majority of the IBV strains isolated from layer flocks affected with SES in SK and AB were Mass type. Similar to EDSV, the Mass type IBV isolate, 15AB-01 was capable of experimentally inducing shell-less eggs in SPF chickens, consistent with the observed association of this isolate with SES in the field outbreaks. Further investigations are warranted to elucidate the pathogenesis of SES and identifying an appropriate vaccine for the control of SES in layer flocks in Western Canada.

## Figures and Tables

**Table 1:** Summary of the tissue screening results for IBV genome (AB = Alberta, SK = Saskatchewan, # = number).

SES Status	Province of Origin	Farm ID	Real-Time PCR Results		# of Samples from Which S1 Nucleotide Sequence Obtained	Sample ID	GenBank Accession #
			# of Samples Screened	# of Samples Positive			
History of SES	AB	1	5	4	4	15AB-01	MH509460
						15AB-06	MH509461
						15AB-10	MH509472
						15AB-14	MH509473
		3	12	3	1	16AB-24	MH509449
		4	9	0	0		
		7	12	1	1	16AB-07	MH509466
	SK	13	74	5	0		
		14	32	6	0		
		16	24	0	0		
		18	12	1	0		
		20	27	3	1	15SK-12	MH509477
		23	15	7	5	15SK-08	MH509448
						15SK21	MH509453
						15SK-23	MH509452
						15SK-26	MH509451
						15SK-27	MH509450
		26	15	2	0		
		27	30	3	0		
Without a history of SES)	AB	2	12	9	5	16AB-13	MH509476
						16AB-15	MH509475
						16AB-20	MH509454
						16AB-22	MH509474
						16AB-28	MH509458
		5	12	0	0		
		6	9	0	0		
	SK	8	15	4	4	15SK-02	MH509465
						15SK-04	MH509462
						15SK-11	MH509470
						15SK-16	MH509471
		9	36	1	0		
		10	48	3	0		
		11	12	1	0		
		12	48	4	1	15SK-03	MH509464
		15	22	2	0		
		17	12	1	0		
		19	27	5	3	15SK-05	MH509463
						15SK-18	MH509469
						15SK-19	MH509459
		21	12	2	1	15SK-29	MH509467
		22	27	9	3	15SK-09	MH509457
						15SK-17	MH509455
						15SK-25	MH509456
		24	15	1	0		
		25	28	2	1	15SK-30	MH509468
	Total		602	79	30		

## Supplementary Materials

The following are available online at http://www.mdpi.com/1999-4915/10/8/437/s1: Production, IBV vaccination history, and the IBV serology data of the study farms are given in the Supplementary Table S1. A complete list of reference IBV sequences used in this study is given in the Supplementary Table S2. Heat map of % amino acid similarity for IBV S1 nucleotide sequences is depicted in the Supplementary Figure S1. Pilot study egg production data is depicted in the Supplementary Figure S2.
