# Supplementary material for: Shell-Less Egg Syndrome (SES) Widespread in Western Canadian Layer Operations Is Linked to a Massachusetts (Mass) Type Infectious Bronchitis Virus (IBV) Isolate
Source: Viruses. 2018 Aug 18;10(8):437. doi: 10.3390/v10080437 (PMC6116215; doi:10.3390/v10080437)
Supplement: Supplementary file 1 [file viruses-10-00437-s001.pdf]

Supplementary Table 1: Flock history and other relevant background of the farms sampled

SES status	Province of origin	Farm ID	Age (weeks)	Breed	Flock size	Drop in production %	% Drop	IBV vaccine used	Method of application	IBV GM Titre
History of SES	AB	1	*	*	8000	93.7 to 46.1	47.6	*	*	*
		3	42	*	6000	*	*	Live	*	*
		4	33	Lohmann Brown	*	*	*	*	*	*
		7	*	*	*	*	*	*	*	*
	SK	13	43	Lohmann LSL	6000	98 to 75	23	Live + Killed	Spray	4891
		14	43	Shaver	26,750	*	*	Live	Spray	5000
		16	25	Bovan	16100	*	*	Live	Spray	*
		18	40	Lohmann	45288	*	*	Live	DW	1109

		20	24	Dekalb	5000	*	*	Live + Killed	*	2576
		23	44	Ross	6373	*	*	Live + Killed	*	8165
		26	46	Lohmann	*	96 to 50	46	Live	Spray	628
		27	36	*	7000	85 to 73	12	Live + Killed	*	6666
			60	*	7000	82 to 75	7	*	*	*
Without a history SES	AB	2	35	Lohmann Brown	6000	N/A		Live + Killed	*	*
		5		Lohmann Brown	*	N/A		*	*	*
		6	56	Isa Brown, Shaver, Bovan	12200, 4000, 2200	N/A		Live + Killed	*	*
	SK	8		*	*	N/A				

9	70	Bovan	*	N/A	Live	Spray	13052
10	46	Bovan	6000	N/A	Live	Spray	1442
11	30	Lohmann LSL	*	N/A	Live	DW	4242
12	33	Lohmann LSL	13200	N/A	Live	Spray	1125
	46			N/A			3630
15	47	Shaver	*	N/A	*		3638
17	40	Bovan	*	N/A	*		4731
19	30	Isa Brown	*	N/A	Live		7381
21	48	Bovan	13770	N/A	Live + killed	DW	7249
	*	H+N		N/A			4698
22	30	Shaver	8000	N/A	Live	DW	4201
	45	Lohmann LSL	17600	N/A	Live	DW	342
	38	Lohmann LSL	17600	N/A	Live	DW	425
24	62	*	*	N/A	*	*	*
25	19	Lohmann LSL	14000	N/A	Live		748
	30			N/A	*		836

Average	39.68	9692.41	21.67	3918.34
Range	18-70	5000-45288	7-46%	342-13052

*Data is not available.
N/A- Not applicable
DW- Drinking water

Supplementary Table 2: List of reference IBV sequences used in the partial S1 sequence analysis.

Reference sequence	GenBank Accession #	Country of origin
Strain UK/7/93	Z83979	United Kingdom
CK/CH/GD/XX12	KJ524641	China
IBV572	KF809797	India
Strain UK/2/91	Z83976	United Kingdom
UK isolate 4/91	JN192154	United Kingdom
Strain 4/91	AF093793	United States
Strain 4/91 vaccine	KF377577	China
NL/L-1449T/04	EF079116	The Netherlands
CK/CH/LSD/07IV	FJ345386	China
Qu16	AF349620	Canada
Qu_mv	AF349621	Canada
Ontario 13-086980-0008	KJ196266	Canada
serotype FL18288	GU393333	United States
Florida 18288	AF027512	United States
Ontario isolate 08-047573	KJ196190	Canada
Ontario 04-42605-CT	KJ196150	Canada
Strain GX-YL130025	KR265092	China
Ontario 01-26070 1	KJ196096	Canada
Connecticut vaccine	KF696629	China
Ontario isolate 02-52451	KJ196128	Canada
vaccine isolate Conn/Bvial2	EU283058	United States
Strain GX-YL130025	KJ999795	China
Strain CK/CH/LLN/111169	KF411040	China
Ontario 10-055626-0025	KJ196194	Canada
IBV Connecticut vaccine	KF696629	China

Ontario 02-52451	KJ196128	Canada
Ontario 01-26070 2	KJ196096	Canada
Ontario 01-30646-A-CT	KJ196098	Canada
Strain H52	AF352315	China
Strain Ma5	KY626045	Brazil
Strain Ma5	AY561713	United States
Isolate B17	KT203557	India
Isolate MDL15-3697	KX529712	United States
Strain ck/CH/LDL/110931	KJ425485	China
Isolate AH07091	FJ829873	China
Strain H120	EU822341	Taiwan
Strain Beaudette	AJ311362	United States
Mass vaccine	EU359657	United States
Isolate UFMG/200	JX182789	Brazil
Ontario 05-43316	KJ196161	Canada
H120 vaccine D	KU736751	Brazil
Ontario 10-101976	KJ196203	Canada
Ontario 13-076490-0005 1	KJ196256	Canada
Isolate MDL_15-1778	KX529719	United States
Isolate 91GX-08X	KC577415	China
Strain ck/CH/LSD/1112150	KJ435286	China
Strain ck/CH/LHLJ/091205	KJ425504	China
Isolate Spain/96/334	DQ064804	Spain
Isolate K446-01	AY257063	Korea
Strain M41	DQ834384	United States
Strain Mass	GQ504724	United States
Strain Mass41	GQ504724	United States
Serotype Holte	GU393336	United States
Serotype Iowa_97	GU393337	United States
Serotype Gray	GU393334	United States

Serotype JMK	GU393338	United States
Strain T6	AF151960	New Zealand
Strain T	AY775779	China
Strain N1-62	DQ490206	Australia
Isolate AL/10928/98	AF510659	United
PDRC_Ark_DPI_38_110087	KX529791	United States
Arkansas Vaccine	GQ504721	United States
Strain Cal557	FJ904715	United States
Isolate GPL8264	GU437864	United States

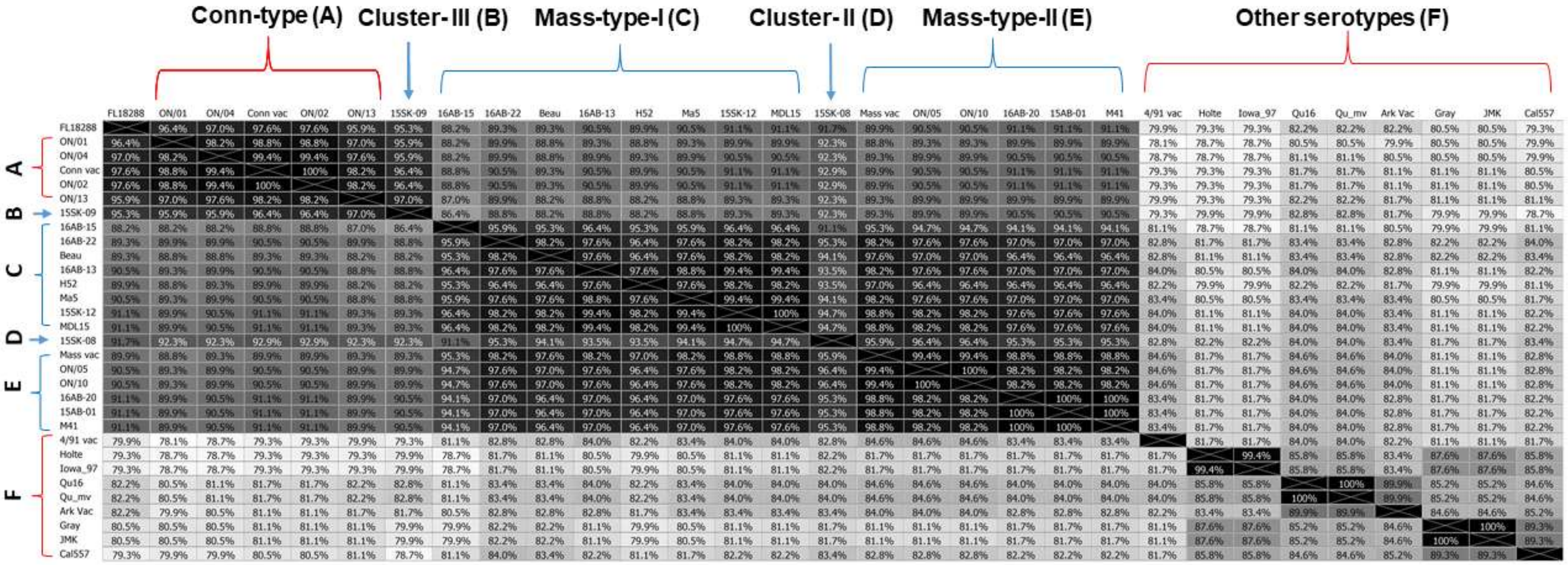

Supplimentary Figure 1. Heat map of amino acid similarity for IBV S1 nucleotide sequences from current IBV isolates are compared with USA and Canadian reference strains. Percentage of the amino acid similarity of the unique current IBVisolates with the selected USA and Canadian reference strains depicted in the similarity matrix along with the heat mapping. Distances among IBV strains are represented by shades of gray. More close and distant strains are shown in darker and lighter shades, respectively.

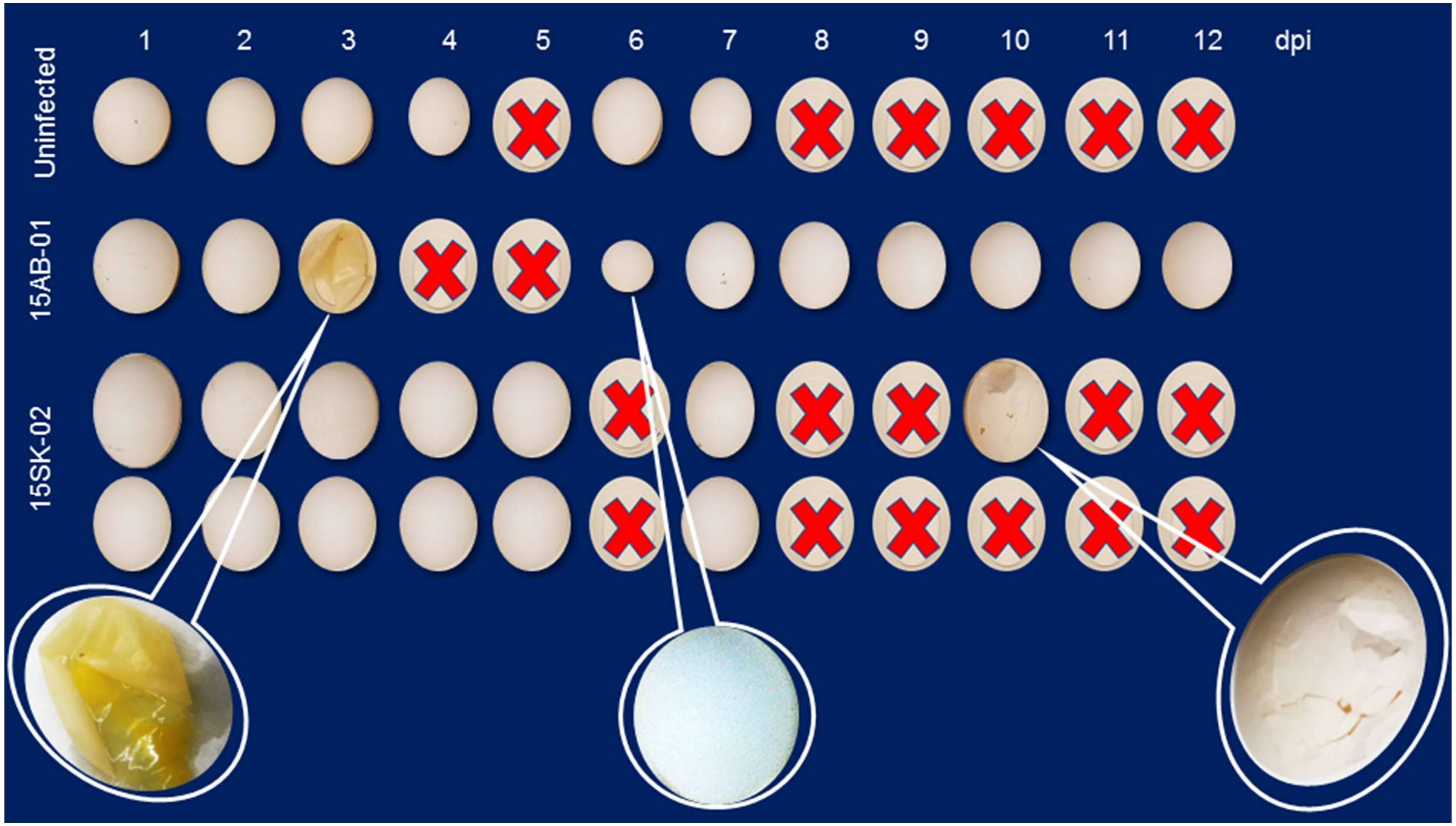

Supplementary Figure 2. Egg production -Pilot SES reproduction study. The gross appearance of the eggs produced during the observation period is shown in the figure for both infected birds and uninfected control. The red X marks indicates no egg production.
